# Reconstruction of the miR-506-Quaking axis in Idiopathic Pulmonary Fibrosis using integrative multi-source bioinformatics

**DOI:** 10.1038/s41598-021-89531-7

**Published:** 2021-06-14

**Authors:** Stevan D. Stojanović, Maximilian Fuchs, Chunguang Liang, Kevin Schmidt, Ke Xiao, Annette Just, Angelika Pfanne, Andreas Pich, Gregor Warnecke, Peter Braubach, Christina Petzold, Danny Jonigk, Jörg H. W. Distler, Jan Fiedler, Thomas Thum, Meik Kunz

**Affiliations:** 1 Institute of Molecular and Translational Therapeutic Strategies (IMTTS), Hannover Medical School, Hannover, Germany; 2 Chair of Medical Informatics, Friedrich-Alexander University (FAU) of Erlangen-Nürnberg, Erlangen, Germany; 3 Fraunhofer Institute of Toxicology and Experimental Medicine (ITEM), Hannover, Germany; 4 Institute of Toxicology and Core Unit Proteomics, Hannover Medical School, Hannover, Germany; 5 Department of Cardiac Surgery, University of Heidelberg, Heidelberg, Germany; 6 Institute of Pathology, Hannover Medical School, Hannover, Germany; 7 Biomedical Research in Endstage and Obstructive Lung Disease Hannover (BREATH), Member of the German Center for Lung Research (DZL), Hannover, Germany; 8 Department of Internal Medicine 3-Rheumatology and Immunology, Universitätsklinikum Erlangen, Friedrich-Alexander University (FAU) of Erlangen-Nürnberg, Erlangen, Germany; 9 Fraunhofer Cluster of Excellence for Immune Mediated Diseases, Hannover, Germany; 10 REBIRTH Center for Translational Regenerative Medicine, Hannover Medical School, Hannover, Germany

**Keywords:** Computational biology and bioinformatics, Diseases, Pathogenesis

## Abstract

The family of RNA-binding proteins (RBP) functions as a crucial regulator of multiple biological processes and diseases. However, RBP function in the clinical setting of idiopathic pulmonary fibrosis (IPF) is still unknown. We developed a practical in silico screening approach for the characterization of RBPs using multi-sources data information and comparative molecular network bioinformatics followed by wet-lab validation studies. Data mining of bulk RNA-Sequencing data of tissues of patients with IPF identified Quaking (QKI) as a significant downregulated RBP. Cell-type specific expression was confirmed by single-cell RNA-Sequencing analysis of IPF patient data. We systematically analyzed the molecular interaction network around QKI and its functional interplay with microRNAs (miRs) in human lung fibroblasts and discovered a novel regulatory miR-506-QKI axis contributing to the pathogenesis of IPF. The in silico results were validated by in-house experiments applying model systems of miR and lung biology. This study supports an understanding of the intrinsic molecular mechanisms of IPF regulated by the miR-506-QKI axis. Initially applied to human lung disease, the herein presented integrative in silico data mining approach can be adapted to other disease entities, underlining its practical relevance in RBP research.

## Introduction

Interstitial lung diseases (ILD) are characterized by progressive extracellular matrix deposition in the inter-alveolar space, leading to irreversible lung scarring^1,2^. The most common form of ILD is idiopathic pulmonary fibrosis (IPF), a disease with poor survival and with limited therapeutic options^1,2^. The only two treatment options for IPF currently approved by the Food and Drug agency (FDA) are Nintedanib and Perfinidone, which prolong survival, but are unable to completely stop or reverse lung fibrosis in a clinical setting^3^. Therefore, elucidating novel mechanisms and molecular targets is essential in the pursuit of improved therapeutic strategies in IPF^1,2,4^.

RNA-binding proteins (RBPs) are an upcoming class of promising molecular targets. RBPs perform a variety of molecular regulations after binding to different forms of RNAs, including RNA splicing, processing, localization and translation^5,6^. RBPs may also exert their function through protein–protein interactions or via enzymatic cores^7^. This multitude of functions was recently translated into the pathophysiological context, multiple RBPs acting as critical orchestrators of abnormal tissue scarring and fibroblast activation, as demonstrated in a setting of heart fibrosis^8^. The RBPs Quaking (QKI) are particularly interesting targets, as rescuing QKI expression has beneficial effects in doxorubicin-induced cardiotoxicity^5^. However, it is unknown if QKI is a therapeutically relevant target in lung fibrosis.

Therapeutic restoration of protein expression can be achieved by inhibiting their intrinsic microRNA (miR) regulators^1,9^. MiRs are small non-coding RNA molecules that inhibit protein expression by binding to mRNA 3′UTR sites, thereby triggering mRNA decay or translational repression. Thus, inhibiting relevant miRs of QKI may be an elegant strategy to restore the loss of protein expression. miR inhibitors are currently in late-preclinical or clinical development for the treatment of heart disease^10,11^.

To explore the possibility of inhibiting miRs to restore QKI expression, we have applied a practical in silico screening approach using multi-sources data information and integrative molecular network bioinformatics. We performed comparative transcriptomics and simulated interaction partners to achieve a deeper insight into the regulatory mechanism of QKI. The generated network served as basis to screen for miRs and drug candidates in silico, which had the potential to act as a molecular off-switch for the QKI-interactome circuit. We have then performed in vitro experiments modelling QKI interactions to confirm the accuracy of the computational predictions.

## Results

### Comparative data mining identifies a loss of QKI expression in IPF

**Figure 1:**
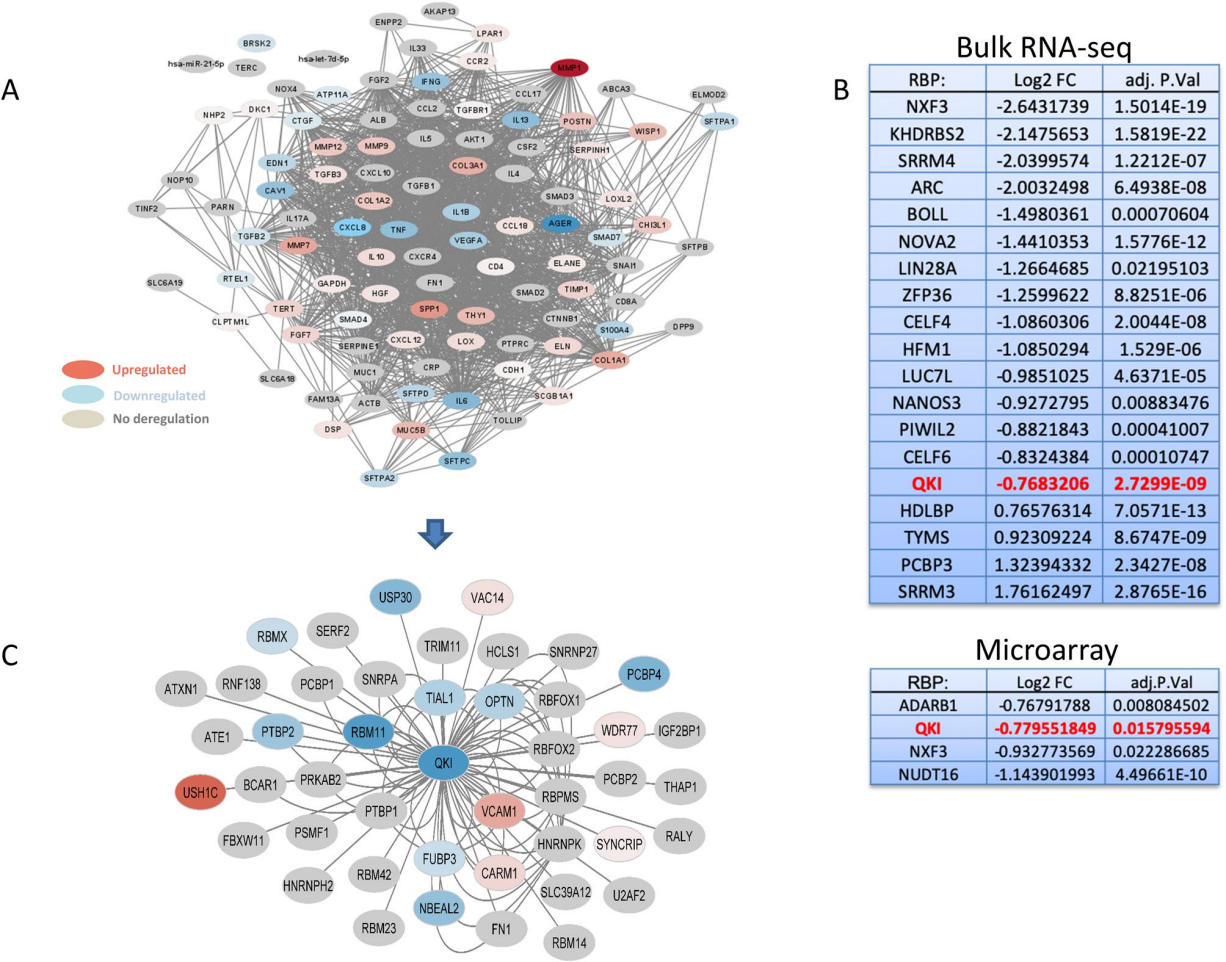
(**A**) IPF-associated disease network showing regulation of known pathophysiological players deregulated in the bulk RNA-Seq (GSE92592) of IPF (n = 20) vs control (n = 19). Nodes represent an interaction partner, edges represent interactions; color scaling shows the deregulation in IPF (red: upregulation, blue: downregulation). (**B**) Extract of annotated RBPs (GO:0003729 = mRNA binding) gene expression in the two independent human IPF datasets (GSE92592, GSE53845). (**C**) QKI-interactome (45 nodes, 87 edges) illustrating interaction partners around QKI and its regulation in a high-throughput RNA-Seq data of human IPF. Nodes represent an interaction partner, edges represent interactions; color scaling shows the deregulation in IPF (red: upregulation, blue: downregulation).

We performed high-throughput data mining using bulk RNA-Seq data (GSE92592) of IPF (n = 20) vs control (n = 19) samples, resulting in 4451 DEGs (logFC > |0.75|; padj < 0.05; Supplementary Table 1). The mapping of the DEGs to the top 100 genes for IPF (= IPF-associated disease network) from the DISEASES^12^ database shows multiple pathophysiological players already known in IPF (Fig. 1A; red: upregulated DEG, blue: downregulated DEG, grey: unregulated). We next screened for RBP-involvement with the disease using 264 annotated RBPs (GO:0003729 = mRNA binding; Supplementary Table 1). Out of them, 19 RBPs had significant deregulation in IPF tissue at the mRNA level (Fig. 1B).

Further analysis with a microarray gene expression dataset (GSE53845)^13^ of human lung tissue samples of IPF patients (n = 40) vs control (n = 8) results in 1206 DEGs (logFC > |0.75|; padj < 0.05; Supplementary Table 1). The mapping against the 264 annotated RBPs (GO:0003729 = mRNA binding; Supplementary Table 1) identifies 4 RBPs (Fig. 1B). Here we found an overlap of QKI and NXF3 in both independent datasets.

We focused on QKI as it has a known role in cardiac fibrosis but not in IPF, and performed a detailed investigation of the QKI regulatory role in human IPF by utilizing a comparative molecular interactome screening approach. The interactome around QKI (= QKI-interactome) showed 45 high-confident interaction partners, in which 15 factors had significant deregulation in the bulk RNA-Seq (Fig. 1C; red nodes are upregulated, blue nodes are downregulated in the bulk RNA-Seq of IPF, GSE92592).

**Figure 2:**
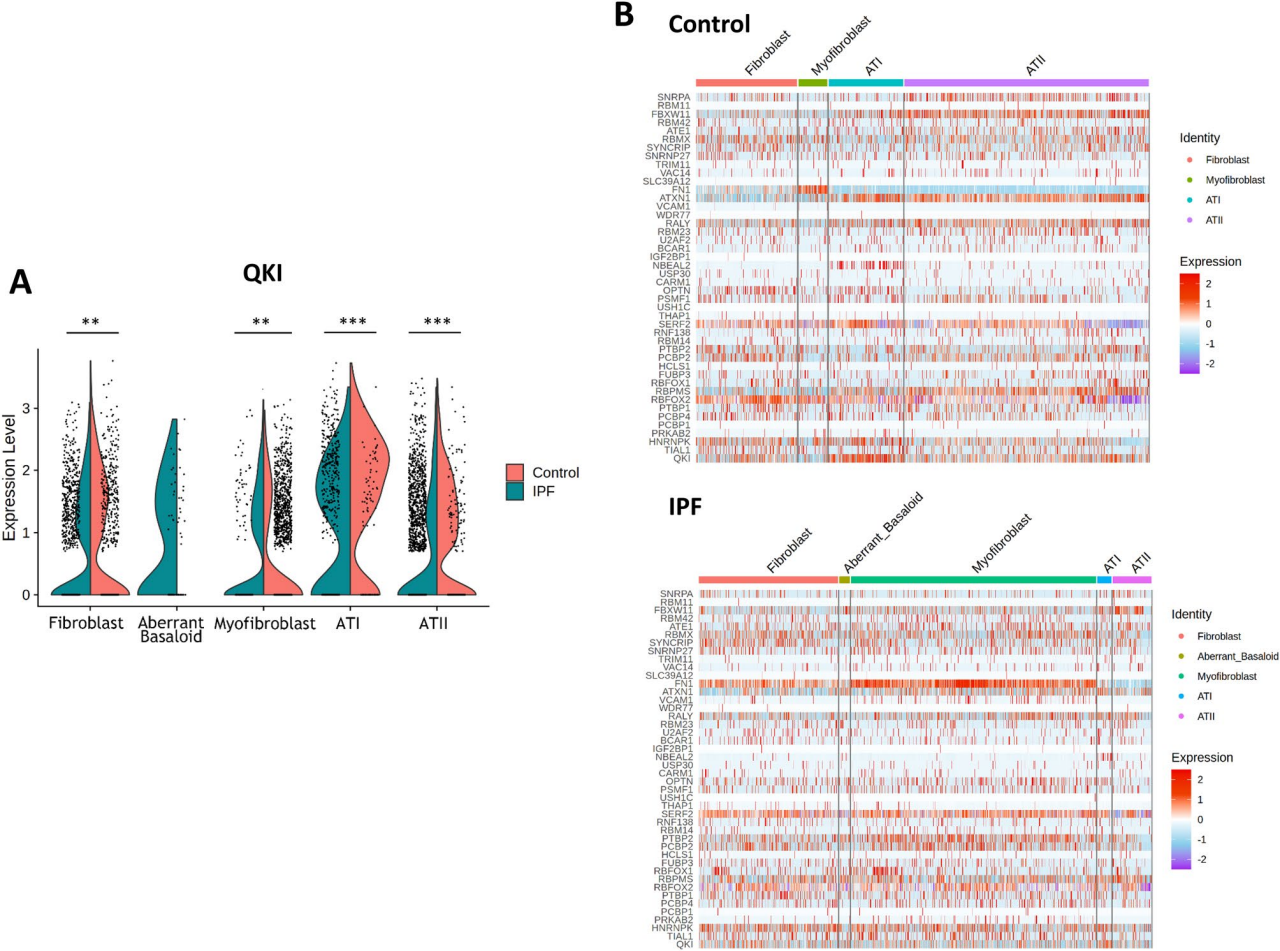
Gene expressions of QKI-interactome members in single-cell RNA-Seq data of IPF (n = 32) and controls (n = 28) (GSE136831^14^; Supplementary Figure 1A shows the identified 39 cell clusters in the dataset). (**A**) Sub-fractions of several pathophysiologically relevant cell populations show low QKI expression patterns in IPF. (**B**) Heatmap illustrating the gene expression of the QKI-interactome in various subpopulations of the single-cell RNA-Seq data in control or IPF cohorts (color scaling shows the gene expression level for given genes and cells in the IPF, red: highest, blue: lowest; see Supplementary Figure 1B–D for all interactome members) (*p < 0.05, **p < 0.01, ***p < 0.001; Welch t-test).

To validate the presence of these interactome nodes of QKI on a single-cell population level, we mined a published single-cell RNA-Seq dataset of IPF (n = 32) and controls (n = 28) (GSE136831^14^; Supplementary Figure 1A shows the UMAP plot of the 39 cell clusters identified in the dataset). Sub-fractions of several cell populations (aberrant basaloid, fibroblast, myofibroblast, type I pneumocyte (ATI) and type II pneumocyte (ATII)) display low QKI expression patterns in IPF (Fig. 2A; Supplementary Figure 1B–D shows all interactome members). Moreover, most members of the QKI-interactome derived from the bulk RNA-Seq data also display deregulation in various subpopulations within the single-cell RNA-Seq data (Fig. 2B). This highlights a broad regulatory role of QKI and its predicted interactome in various cell types in IPF.

### miR-binding screening reveals miR-506-3p as a critical regulator of the QKI-interactome and pro-fibrotic factors

We then proceeded to evaluate the susceptibility of the QKI-interactome to miR-regulation. The miR-interaction screening of the reconstructed IPF-related molecular QKI-interactome resulted in an extended QKI-miR-interactome of 240 nodes and 480 edges (Supplementary Figure 2). Next, we calculated for each node in this network the centrality value in order to analyze its regulatory network effect (Supplementary Table 1). Here, QKI showed the highest network centrality value (0.70), whereas miR-506-3p had the highest network centrality value (0.003) directly interacting with QKI and three downregulated targets (logFC: NBEAL2 = − 0.44, PTBP2 = − 0.40, TIAL1 = − 0.31) from the bulk RNA-Seq of IPF (GSE92592). This finding indicated the miR-506 as a potent repressor of the QKI-interactome and thus a relevant regulatory module in the IPF.

**Figure 3:**
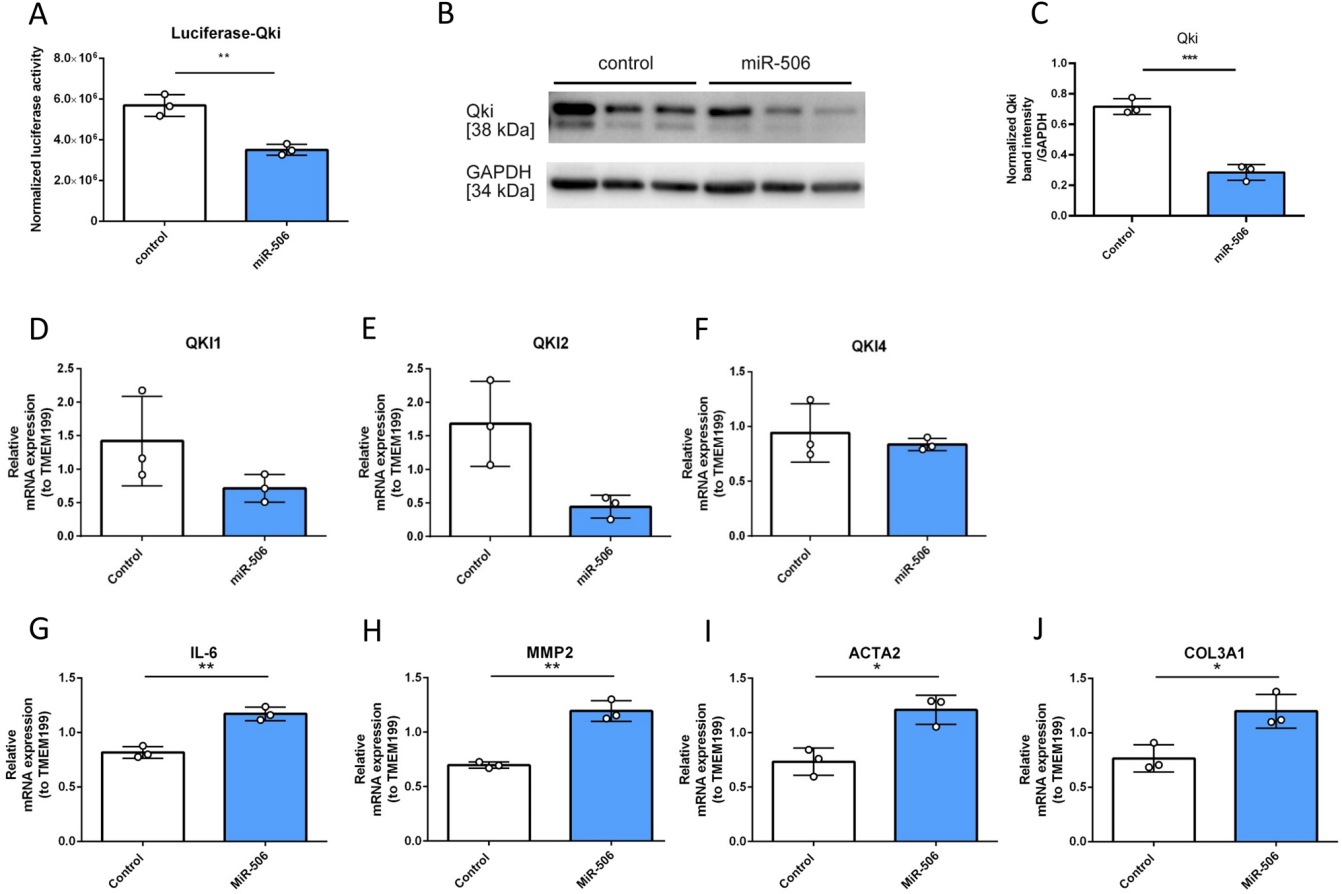
(**A**) Validation of miR-506-3p binding to human QKI 3′-UTR via luciferase assay. (n = 3 experiments). (**B**,**C**) QKI is downregulated via miR-506 in human lung fibroblasts after 48 h of miR transfection. QKI (exposure 145.708 s) and GAPDH (exposure 36.664 s) detection via Western Blot after 48 h of overexpression (representative Western Blot) in vitro and statistical evaluation. (n = 3 experiments). Gene expression levels of (**D**–**F**) various QKI mRNA variants and (**G**–**J**) factors relevant for fibrosis (RT-qPCR) (*p < 0.05, **p < 0.01; t-test).

Bioinformatics predicted miR-506 binding to QKI 3′-UTR was then validated in a luciferase reporter system mimicking miR:mRNA binding (Fig. 3A, Supplementary Figure 3). We evaluated the results of our in silico screening by modulating endogenous miR-506 in human lung fibroblasts (MRC5). Indeed, we confirmed QKI repression via miR-506 in this cell culture model after 48 h (Fig. 3B,C). miR-506 was also predicted in silico to bind to 3′UTR segments of all QKI mRNA isoforms using TargetScan v7.2^15^. We have thus evaluated the effect of miR-506 on various QKI mRNA splicing variants (Fig. 3D–F). QKI1, QKI2 but not QKI4, exhibited trends towards downregulation.

We then evaluated the relevance of these effects in a cellular setting by measuring gene expression levels of various factors relevant for fibrosis. After treatment with miR-506, MRC5 lung fibroblasts showed significantly higher expression levels of IL6, MMP2, ACTA2 and COL3A1, indicating the pro-fibrotic biological contribution of the miR-506-QKI axis (Fig. 3G–J).

**Figure 4:**
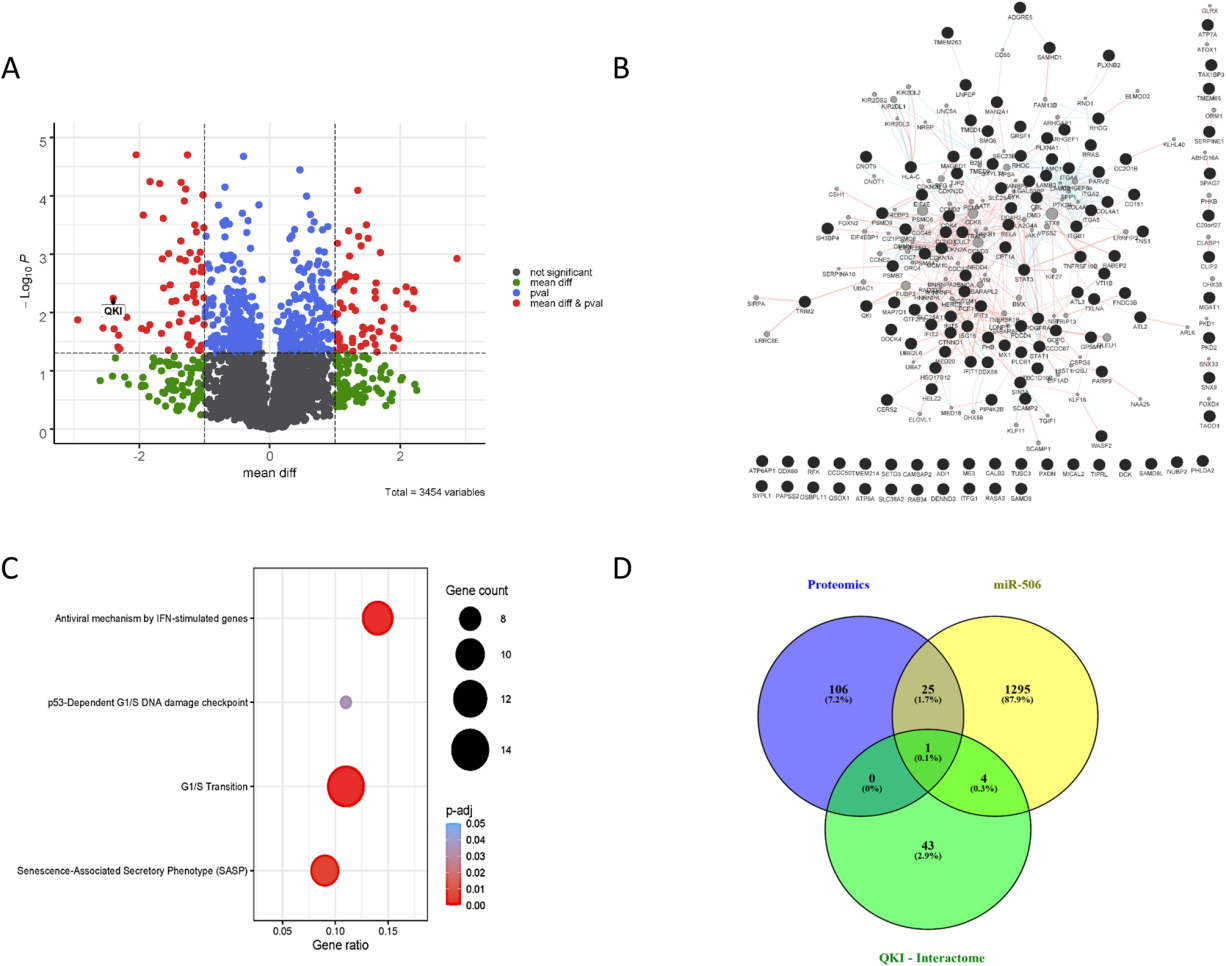
(**A**) Volcano plot of proteomics analysis after MRC5 treatment with miR-506. (**B**) Interaction network from proteomics. The 132 deregulated proteins of the miR-506-modulated proteomics resulted in a miR-506-proteome-interactome of 229 nodes and 578 edges. Nodes represent an interaction partner, edges represent interactions. (**C**) Functional enrichment analysis of proteomics data. Bubble size illustrates the ratio between the proteins connected to a process and the overall query size. Color scaling according to the adjusted *p*-value (Supplementary Table 1 for whole functional enrichment analysis using g:Profiler). (**D**) Mapping of the QKI-interactome, proteomics dataset and miR-506-interaction partners showed QKI as overlapping regulator.

To further validate the importance of the miR-506-QKI interaction, we performed proteomics data on human lung fibroblasts after control or miR-506 treatment (Fig. 4A; Supplementary Figure 3B, Supplementary Table 1). QKI was again one of the strongly repressed targets. In terms of the global effects of miR-506 treatment, we performed a highly-resolving proteome analysis of miR-506-modulated lung fibroblasts, by extending the 132 proteins by direct interaction partners reconstitute a miR-506-proteome-interactome of 229 nodes and 578 edges (Fig. 4B; Supplementary Table 1).

Functional enrichment analysis of the 229 nodes of the interactome found 226 significantly enriched functional processes and pathways associated with fibrosis-relevant processes such as Senescence-Associated Secretory Phenotype (SASP) and p53-Dependent G1/S DNA damage checkpoint (Fig. 4C; Supplementary Table 1). Mapping of the proteomics dataset to the QKI-interactome and miR-506-interaction partners showed QKI as a common regulatory node (Fig. 4D), pointing towards QKI as the likely key hub for miR-506 downstream effects. In summary, these network predictions underline a miR-cluster regulation around the miR-506-QKI axis in human IPF.

## Discussion

Fibrotic lung diseases, including IPF, are complex disturbances with a variety of processes that are so far unexplored, in particular in terms of detailing underlying interplay of proteins and noncoding RNAs. Integrative bioinformatics approaches have significant promise in shedding a new light on otherwise obscured processes. We have demonstrated the feasibility of computational methodology to unravel understudied processes and potential pharmacological targets in lung fibrosis. Our approach was based on previously published clinical IPF bulk RNA-Seq and microarray data including a substantial patient cohort^13,16^. We chose to use bulk RNA-Seq as a basis for further analysis, as this technology is widely used and best standardized in various settings. However, the bulk tissue-omics have significant limitations, as the expression profiles of rare but significant cell populations may be lost between the noise of more common cell types. By mining data from a single cell RNA-Seq dataset from Adams et al.^14^, we could in fact show fractions within various cell populations with low QKI expression. Multi-source data mining and comparative bioinformatics analysis may thus allow the extraction of underappreciated targets from bulk RNA-Seq data, as the less dominant signatures may be inferred from interaction partner screenings.

In the context of IPF, we have detected the loss of QKI expression, which is a significant event in various diseases such as the heart^5^. In IPF, QKI showed a loss in certain fractions of various cell types. Of note, this was most prominent in multiple cell types which are highly relevant for IPF pathophysiology (myofibroblast, fibroblast, aberrant basaloid, ATI, ATII). We discovered a novel molecular regulatory miR-506-QKI axis in human IPF in silico, which was directly validated in vitro, underlining the practical relevance and translational potential of our in silico approach in RBP research. The miR-506-QKI axis might be a promising and biologically relevant target, as supported by pro-fibrotic gene expression and fibrosis-relevant processes^16,17^ in lung fibroblasts in vitro.

It is possible that miR-506 exerts some of its effects through other members of its targetome, as all miRs often have thousands of targets^18,19^. However, QKI was consistently repressed in different datasets and cell sub-populations used in this study. The mechanism of repression is likely mostly due to repression of multiple QKI mRNA isoforms (QKI1, QKI2), as individual mRNA isoforms showed a trend towards degradation. QKI loss at the protein level mediated by miR-506 is thus likely based on concomitant mRNA degradation and translational repression^18,19^ of several QKI isoforms. This would likely trigger a complex set of events ultimately leading to a pro-fibrotic phenotype, given the multitude of roles and localizations of various QKI isoforms^20,21^. A loss of QKI-abundant ATI and ATII cells may have also contributed to the overall loss of QKI in the bulk omics data.

Loss of QKI is correlated with a poor prognosis in lung cancer. Of note, IPF patients suffer from a pronounced risk of developing lung cancer and there may be a connection between low QKI in IPF and this important clinical risk^22^. miR-506 was also reported as a tumor-suppressor^23^. It thus may be necessary to inhibit this miR in a low-dose approach or short-term treatment to avoid oncogenic transformation. Interestingly, miR-506 comprises a family of miRs expressed from the X-chromosome enhancing the complexity of their interplay and molecular targets^24^. It remains to be seen if inhibiting this miR or one of its family members provides clinical benefit in IPF.

In conclusion, we have confirmed a high potential of QKI-miR-506 axis involvement in lung fibrosis applying a practical in silico approach enabling the assessment of RBP regulation from any high-throughput dataset available in literature or clinical material. We herein emphasized the applicability of this bioinformatics approach to human lung diseases, although it can be adapted to other disease entities in similar manner.

## Methods

### Integrative bioinformatics transcriptome and proteome profiling analysis

Gene expression data of IPF (n = 20) vs. control (n = 19) were obtained from GEO database (GSE92592^16^). Bioinformatics analysis of raw read counts was performed using the built-in graphical user interface of our developed R package tRomics^9^. Differential expression analysis was performed using DESeq2^25^. Genes were considered differentially expressed (DEGs) with logarithmic fold chance (logFC) > |0.75| and false discovery rate (FDR)-adjusted p-value < 0.05. DEGs were screened against the 264 GO annotated RBPs (GO:0003729 = mRNA binding; Supplementary Table 1; accessed Jan 2021). For the reconstruction of the IPF-associated disease network we selected the top 100 genes for IPF from the DISEASES gene association database^12^ using the Cytoscape StringApp^26^.

As additional validation dataset we used a gene expression dataset (GSE53845)^13^ of human lung tissue samples of IPF patient (n = 40) vs control (n = 8) (logFC > |0.75|; padj < 0.05). For this, raw data was collected from GEO database using GEOquery 2.56.0, and differential expression analysis was performed using limma 3.44.3.

For further molecular regulatory network analysis, RBPs were selected based on the overlap in the bulk RNA-Seq and microarray datasets, association with fibrosis, leading to QKI as top candidate.

For the reconstruction of the molecular QKI-interactome, we used human interactome data from the International Molecular Exchange (IMEx) database^27^ (accessed Oct 2020) and miR-interaction partners from the TargetScanHuman^15^ implemented in the Cytoscape (v 3.8.1) plugin CyTargetLinker (v 4.1.0)^28^. Statistical regulatory network analysis was performed using Cytoscape plugin NetworkAnalyzer^29^ (v 4.3.1).

Single-cell RNA-Seq data of IPF (n = 32), COPD (n = 18) and controls (n = 28) were downloaded from GEO database (GSE136831^14^; in total there are 312,928 cells and 45,947 gene features). Among them, cells of high quality with mitochondrial gene content lower than 10%, feature total number higher than 500 and lower than 3000, RNA count number lower than 10,000 were extracted. Seurat package^30^ (version 3.2.3) were further applied to analyze the selected 198,992 cells to achieve 39 cell clusters for all the three libraries (IPF, Control, COPD). We extracted five cell-types (aberrant basaloid, fibroblast, myofibroblast, type I pneumocyte (ATI) and type II pneumocyte (ATII)) from two groups (IPF, Control) to further investigate the gene expression of the QKI-interactome in the IPF background.

We further analyzed a proteomics dataset of miR-506-modulated MRC-5 lung fibroblasts. Proteins which showed significant deregulation (pV < 0.05; mean difference > |1.0|) were used for generating a miR-506-proteome-interactome using interaction partners from literature and databases by applying the Cytoscape (version 3.8.1) plugin GeneMANIA version 3.5.1^31^. The resulting interactome was further analysed for functional enrichment using g:Profiler web tool^32^. Selected significantly enriched processes (adjusted p-value < 0.05) were plotted using ggplot2 package version 3.3.2^33^ in R (version 4.0.2).

### Cell culture and miR transfection

Cell culture and miR transfection were performed similar to our published experiences^1,34^. Human fibroblasts originating from fetal lungs (MRC-5) were acquired from ATCC. The cells cultivated in Dulbecco's Modified Eagle Medium (Thermo Fisher, 11965084), with the addition of 10% FBS and 1% penicillin–streptomycin (Sigma Aldrich). Transient transfection of cells was performed at a confluence of 60–70%, 24 h after seeding. Final concentration of miRVana oligonucleotides (Thermo Fisher) was 30 nM for miR control or miR-506-3p. miRVana mimics and Lipofectamine 2000 (Invitrogen) were kept separate with Opti-MEM I medium (Invitrogen) for 5 min, then mixed and incubated for 20 min. The transfection reaction was performed in serum-free conditions, after which change of medium was performed after 4 h. Cells were processed for further analysis after 48 h.

### Protein isolation and Western blot

Protein extraction and Western blotting was performed similarly as in our previous work^1^. Cells were washed with PBS, after which total protein was isolated from cell pellets using a cell lysis buffer (Biorad). Samples were pipetted onto polyacrylamide gels for electrophoretic band separation and transferred to a nitrocellulose membrane (Biorad). Blocking of membranes was performed by incubation for 1 h in 5% milk solution. The membranes were incubated with the primary antibody overnight. After rinsing, the membranes were exposed to IgG-HRP secondary antibodies, ultimately being washed again and visualized via the enhanced chemiluminescence (ECL) reagent (Biorad). Antibodies used for detection of QKI and GAPDH were as following: rabbit-anti-Qki (Sigma HPA019123) and mouse-anti-GAPDH (Abcam ab8245).

### Luciferase reporter assay for miR-506-3p binding at human QKI 3′-UTR

The luciferase reporter assay was performed as published^34^. In brief, the QKI 3′-UTR fragment was PCR-amplified from human cDNA utilizing SpeI and HindIII restriction sites integrated to primer sequences. After restriction digest, the fragment was subcloned to pmiR report plasmid (Promega) and sequenced for appearance of correct miR binding site. The generated reporter plasmid was then co-transfected with 30 nM control miR or miR-506-3p and beta-Gal expressing plasmid for normalization (Promega) into HEK293 cells for 24 h. Validation of luciferase and beta-galactosidase activity was then performed.

### RNA isolation and RT-qPCR

RNA isolation was performed with the miRNeasy Mini Kit (Qiagen) by following manifacturer recommendations. Gene expression of IL6, MMP2, COL3A1 and ACTA2 was conducted after reverse transciption with the iScript cDNA Synthesis Kit (BioRad) and quantification via the iQ SYBR Green Supermix (Biorad), with the following primers^35^: IL-6 (Forward 5′-GGCACTGGCAGAAAACAACC-3′; Reverse 5′-GCAAGTCTCCTCATTGAATCC-3′; Eurofins), MMP2 (QT00088396, Qiagen Quantitect), ACTA2 (Forward 5′-CCTGACTGAGCGTGGCTATT3-3′; Reverse 5′-GATGAAGGATGGCTGGAACA-3′), COL3A1 (QT0036598, Qiagen Quantitect), QKI1 (Forward 5′-CAGCCCTTGCCTTTTCTCTTGC-3′; Reverse 5′-ATAGGTTAGTTGCCGGTGGC-3′; Eurofins), QKI2 (Forward 5′-GGGCCTGAAGCTGGGTTAAT-3′; Reverse: 5′-GCCTTTCGTTGGGAAAGCCA-3′; Eurofins), QKI4 (Forward 5′-GGAGCTTGCGATTCTGAATG-3′; Reverse: 5′-TAACCCCATGGGGAGAAGAAC-3′; Eurofins). Data was normalized to TMEM199 as the housekeeping gene (Forward 5′-CACCAGCATCTGAGAGAAAGG-3′; Reverse 5′-CCGTGGAGGCTTCACAAC-3′; Eurofins). miR-506-3p detection was performed with a specific TaqMan miR assay, as specified by the manufacturer (Thermo Fisher, 001050).

### LC–MS based proteomics

LC–MS based proteomics was performed as described previously^35^. In short, whole cell lysates from MRC-5 fibroblast controls or miR-506 transfected cells were generated, and proteins cysteine residues were alkylated with acrylamide. Proteins were separated on pre-casted SDS gels (BioRad) and stained with Coomassie. Lanes were sliced into 4 pieces and after destaining proteins were in-gel digested with trypsin overnight. Resulting peptides were extracted and analyzed in a LC–MS system consisting of a nanoLC and an Orbitrap MS (RSLC, LTQ Orbiotrap Velos, both Thermo Fisher Scientific). Raw data were processed with MaxQuant software (version 1.5.3.30) and peptides were searched against reviewed human entries of the UniProt database via the Andromeda search engine. A FDR of 0.01 on peptide and protein level was set. Data were analyzed in Perseus (version 1.5.2.6) and if applicable, a two sided two-sample Student’s t-test was applied.

## Supplementary Information

Supplementary Information 1.

Supplementary Information 2.

Supplementary Information 3.

## Data Availability

All data are available in the manuscript and as supplement online.
