# Supplementary material for: Reconstruction of the miR-506-Quaking axis in Idiopathic Pulmonary Fibrosis using integrative multi-source bioinformatics
Source: Sci Rep. 2021 Jun 14;11:12456. doi: 10.1038/s41598-021-89531-7 (PMC8203802; doi:10.1038/s41598-021-89531-7)

Reverse engineering of the miR-506-Quaking axis in Idiopathic Pulmonary Fibrosis using integrative multi-source bioinformatics

Stevan D. Stojanović[1]*, Maximilian Fuchs[2]*, Ke Xiao[1,3], Annette Just[1], Andreas Pich[4], Jörg H.W. Distler[5], Jan Fiedler[1,3*,#,] Thomas Thum[1,3,6*,#,] Meik Kunz[2*,#]
[1]Institute of Molecular and Translational Therapeutic Strategies (IMTTS), Hannover Medical School, Hannover, Germany
[2]Chair of Medical Informatics, Friedrich-Alexander University (FAU) of Erlangen-Nürnberg, Erlangen, Germany
[3]Fraunhofer ITEM
[4]Institute of Toxicology and Core Unit Proteomics, Hannover Medical School, Hannover, Germany
[5]Friedrich-Alexander University (FAU) of Erlangen-Nürnberg, Department of Internal Medicine 3 - Rheumatology and Immunology, Universitätsklinikum Erlangen, Erlangen, Germany;
[6]REBIRTH Center for Translational Regenerative Medicine, Hannover Medical School, Hannover, Germany
* shared first and last authorships
#Correspondence: meik.kunz@fau.de; Tel.: +49-9131-85-26767; Fax:
++49-9131-85-26754; Thum.Thomas@mh-hannover.de, Tel.: +49-511-532-5272,
Fax: ++49-511-532-5274; Fiedler.Jan@mh-hannover.de, Tel.: +49-511-532-5276

Supplementary Figure 1

Original Western blot images (from Figure 2)

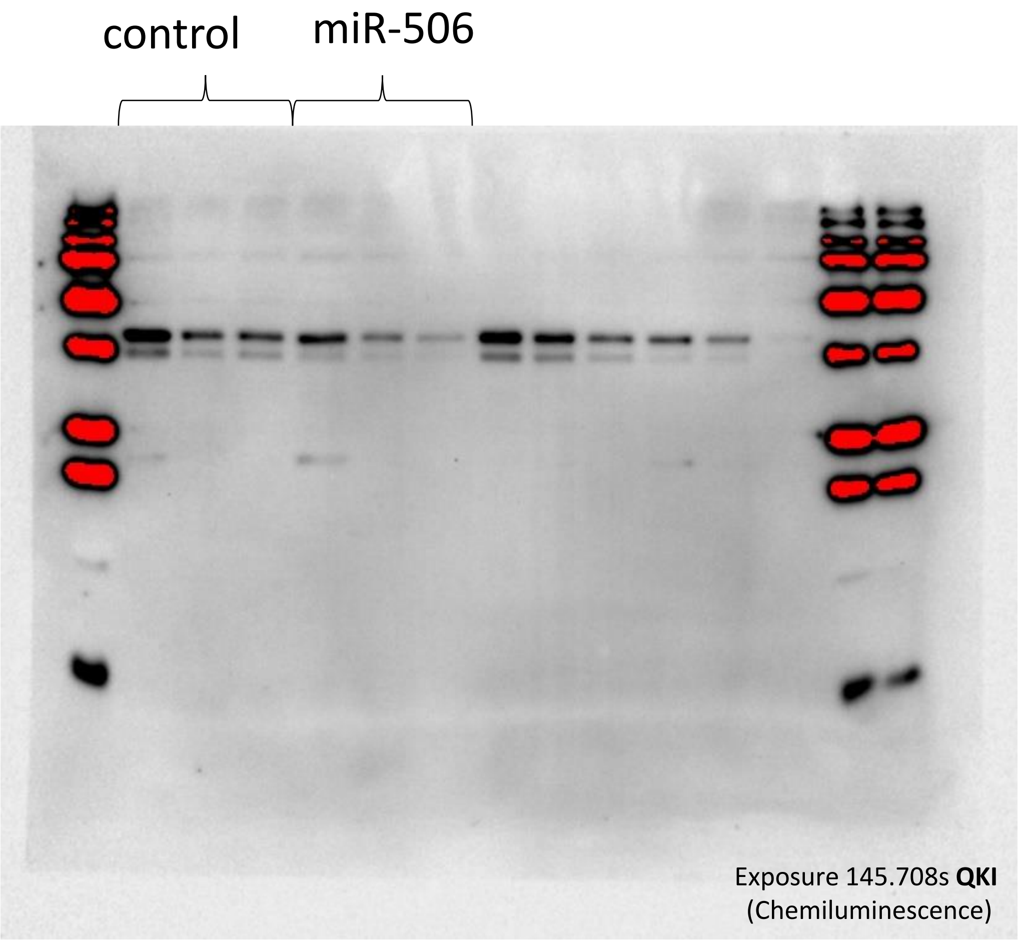

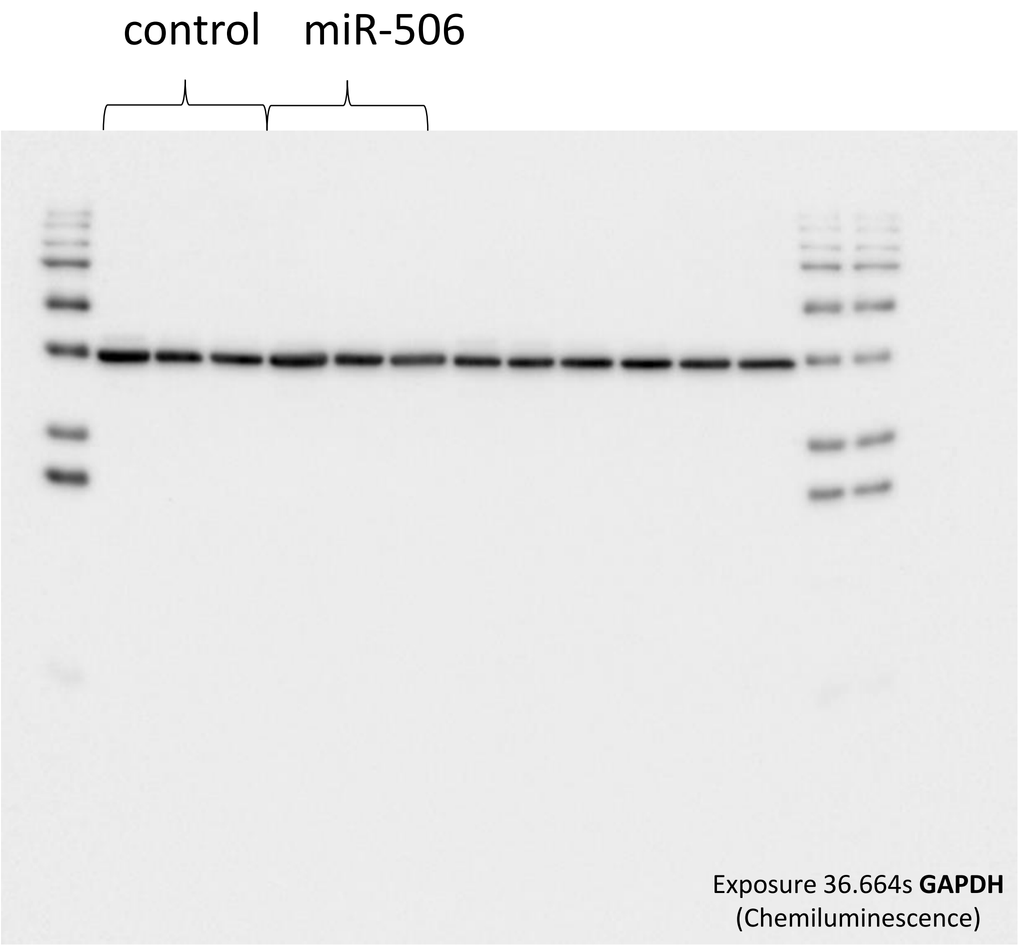

Supplement: Supplementary file 1 — Supplementary Information 1. [file 41598_2021_89531_MOESM1_ESM.pdf]
