# Supplementary material for: Reconstruction of the miR-506-Quaking axis in Idiopathic Pulmonary Fibrosis using integrative multi-source bioinformatics
Source: Sci Rep. 2021 Jun 14;11:12456. doi: 10.1038/s41598-021-89531-7 (PMC8203802; doi:10.1038/s41598-021-89531-7)
Supplement: Supplementary file 3 — Supplementary Information 3. [file 41598_2021_89531_MOESM3_ESM.pptx]

## Slide 1
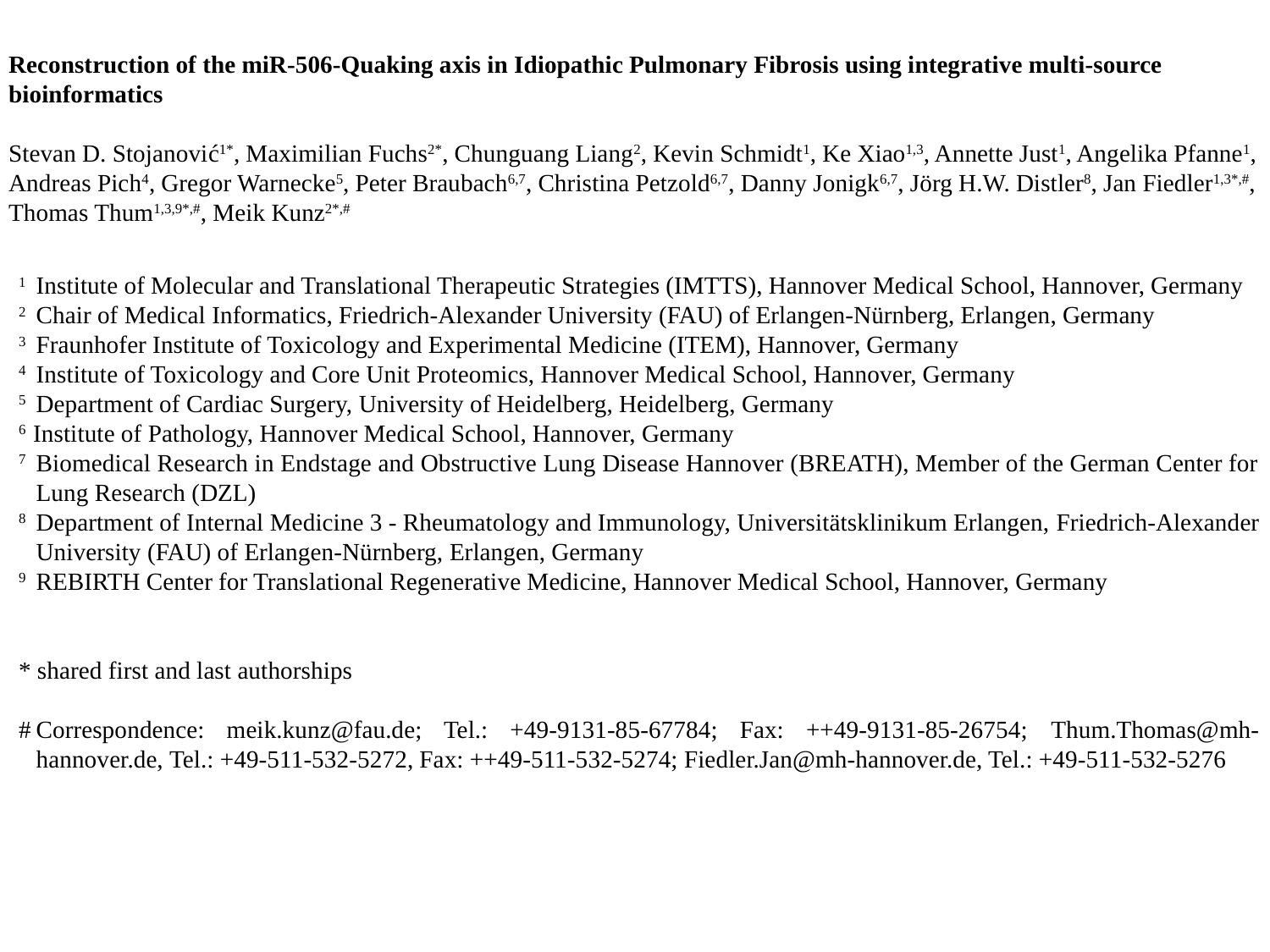

Reconstruction of the miR-506-Quaking axis in Idiopathic Pulmonary Fibrosis using integrative multi-source bioinformatics
Stevan D. Stojanović1*, Maximilian Fuchs2*, Chunguang Liang2, Kevin Schmidt1, Ke Xiao1,3, Annette Just1, Angelika Pfanne1, Andreas Pich4, Gregor Warnecke5, Peter Braubach6,7, Christina Petzold6,7, Danny Jonigk6,7, Jörg H.W. Distler8, Jan Fiedler1,3*,#, Thomas Thum1,3,9*,#, Meik Kunz2*,#
1	Institute of Molecular and Translational Therapeutic Strategies (IMTTS), Hannover Medical School, Hannover, Germany
2	Chair of Medical Informatics, Friedrich-Alexander University (FAU) of Erlangen-Nürnberg, Erlangen, Germany
3	Fraunhofer Institute of Toxicology and Experimental Medicine (ITEM), Hannover, Germany
4	Institute of Toxicology and Core Unit Proteomics, Hannover Medical School, Hannover, Germany
5	Department of Cardiac Surgery, University of Heidelberg, Heidelberg, Germany
6 Institute of Pathology, Hannover Medical School, Hannover, Germany
7	Biomedical Research in Endstage and Obstructive Lung Disease Hannover (BREATH), Member of the German Center for Lung Research (DZL)
8	Department of Internal Medicine 3 - Rheumatology and Immunology, Universitätsklinikum Erlangen, Friedrich-Alexander University (FAU) of Erlangen-Nürnberg, Erlangen, Germany
9	REBIRTH Center for Translational Regenerative Medicine, Hannover Medical School, Hannover, Germany
* shared first and last authorships
#	Correspondence: meik.kunz@fau.de; Tel.: +49-9131-85-67784; Fax: ++49-9131-85-26754; Thum.Thomas@mh-hannover.de, Tel.: +49-511-532-5272, Fax: ++49-511-532-5274; Fiedler.Jan@mh-hannover.de, Tel.: +49-511-532-5276

## Slide 2
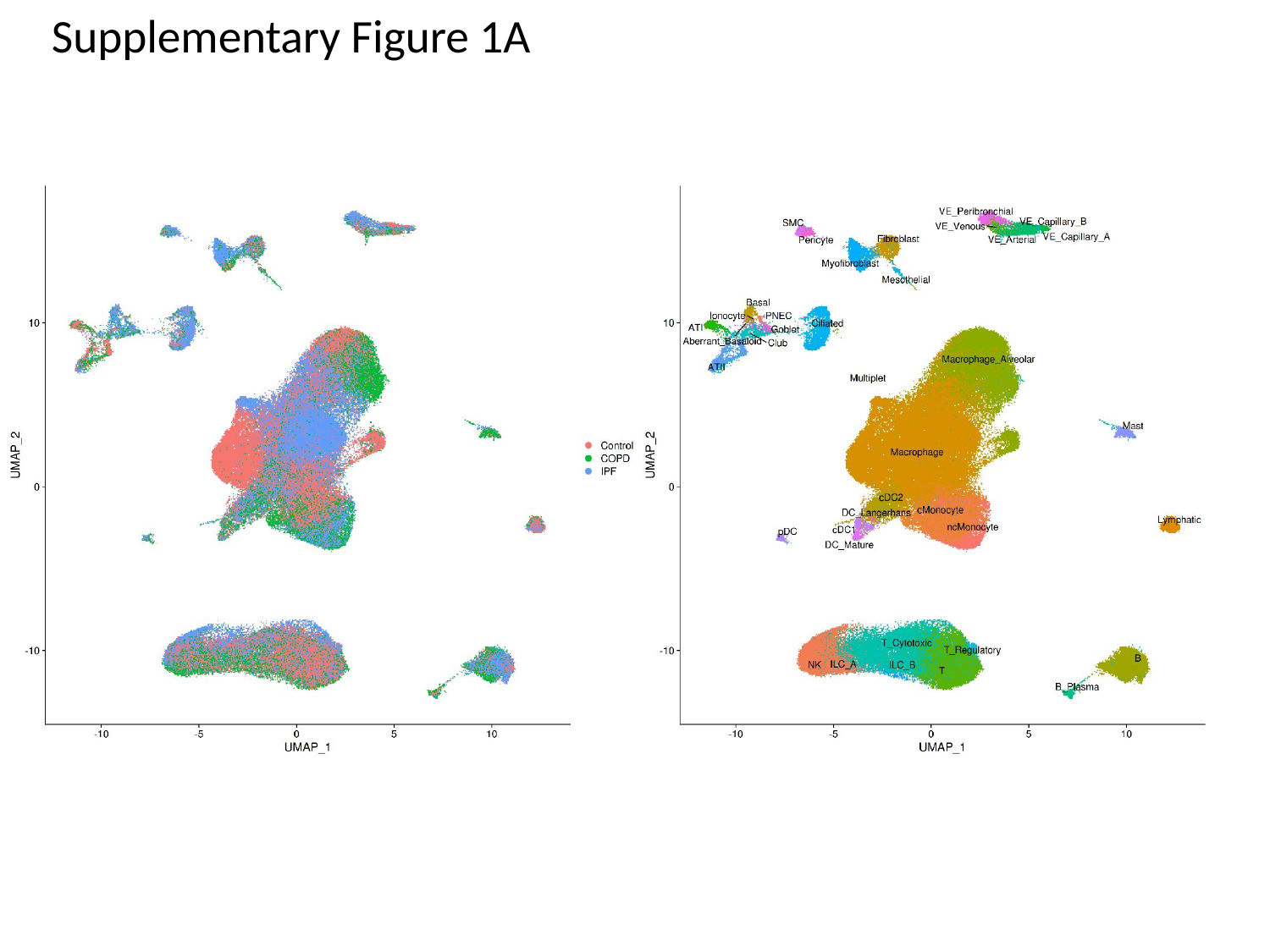

Supplementary Figure 1A

## Slide 3
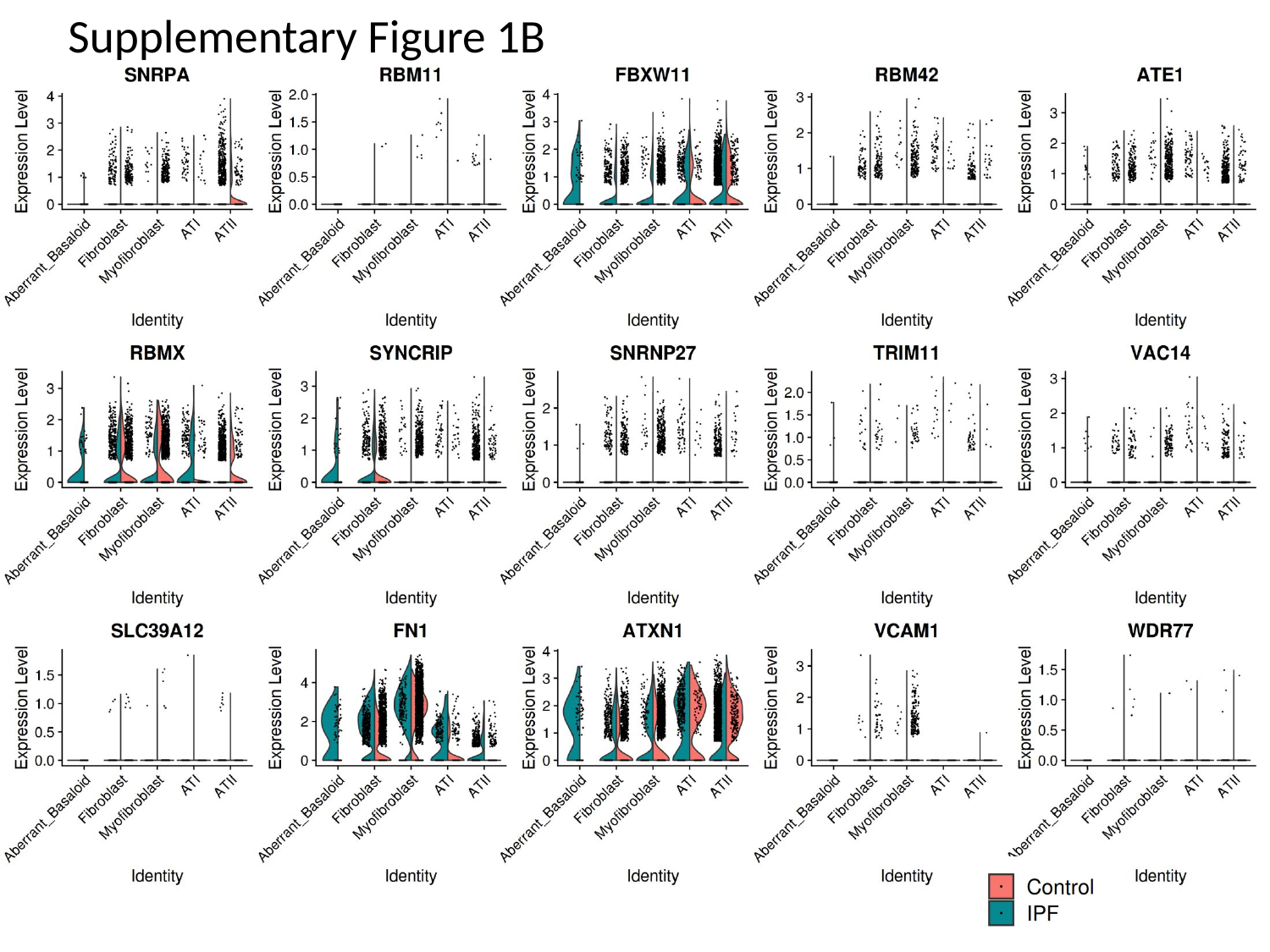

Supplementary Figure 1B
#

## Slide 4
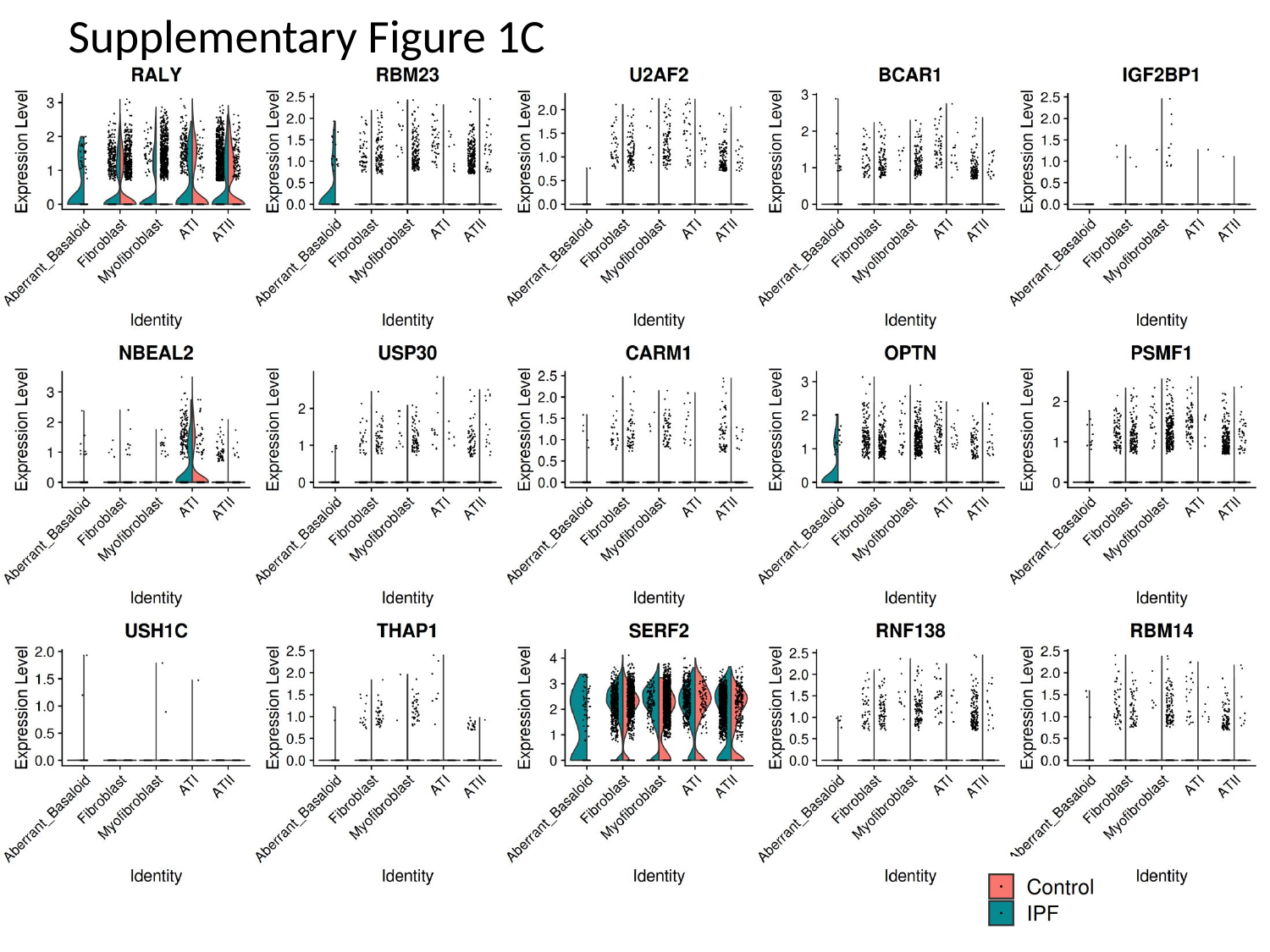

Supplementary Figure 1C

## Slide 5
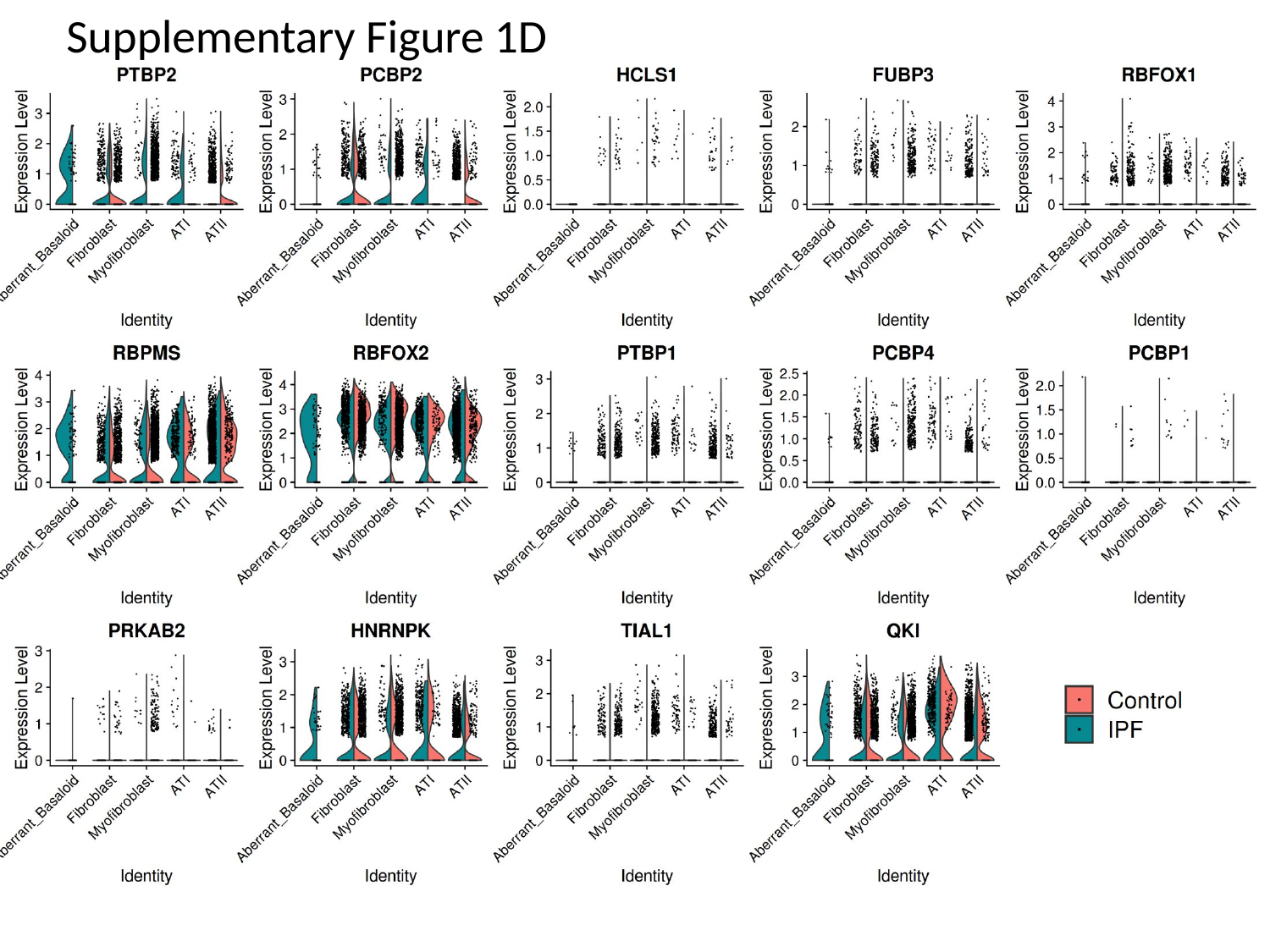

Supplementary Figure 1D

## Slide 6
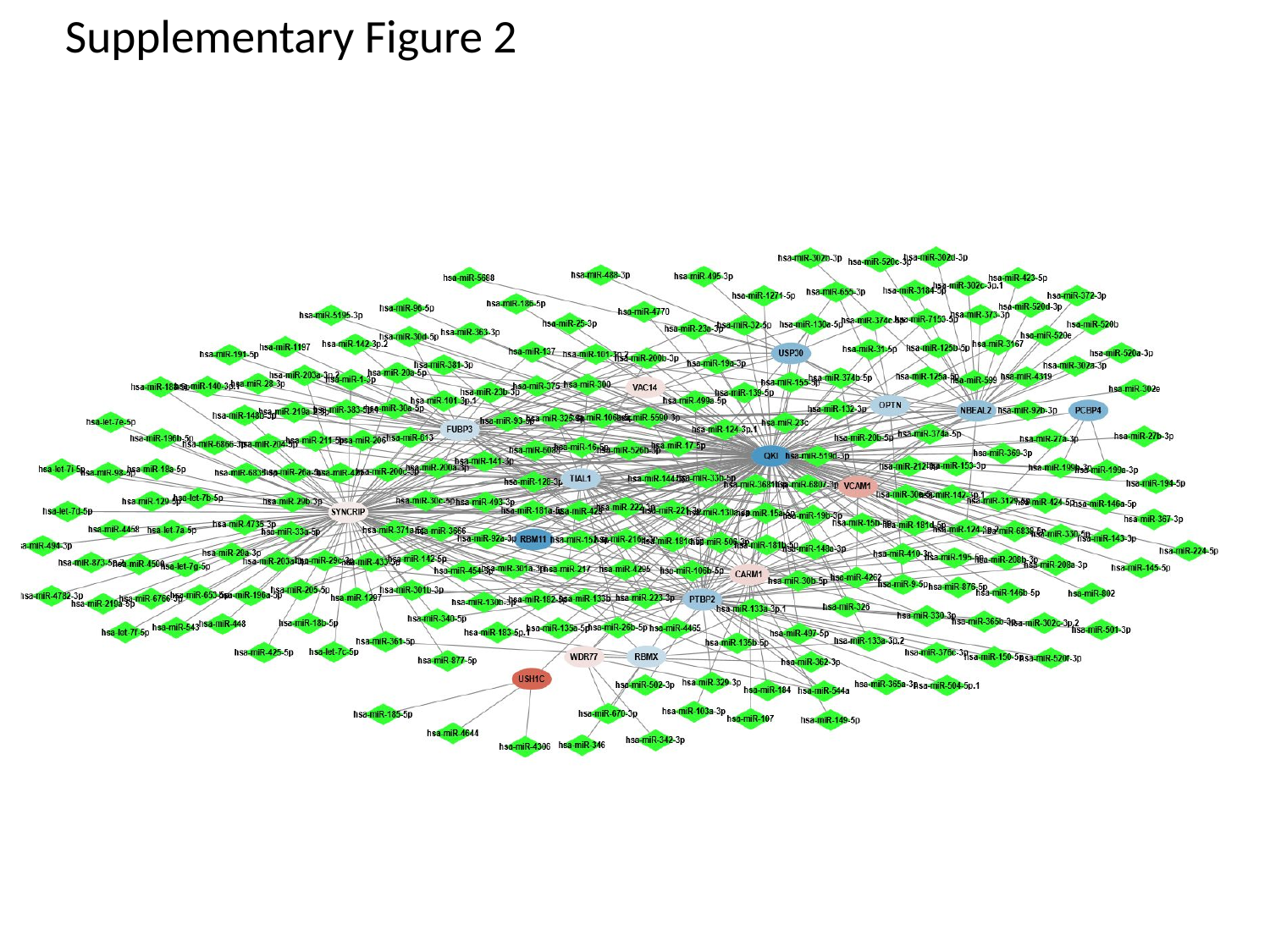

Supplementary Figure 2

## Slide 7
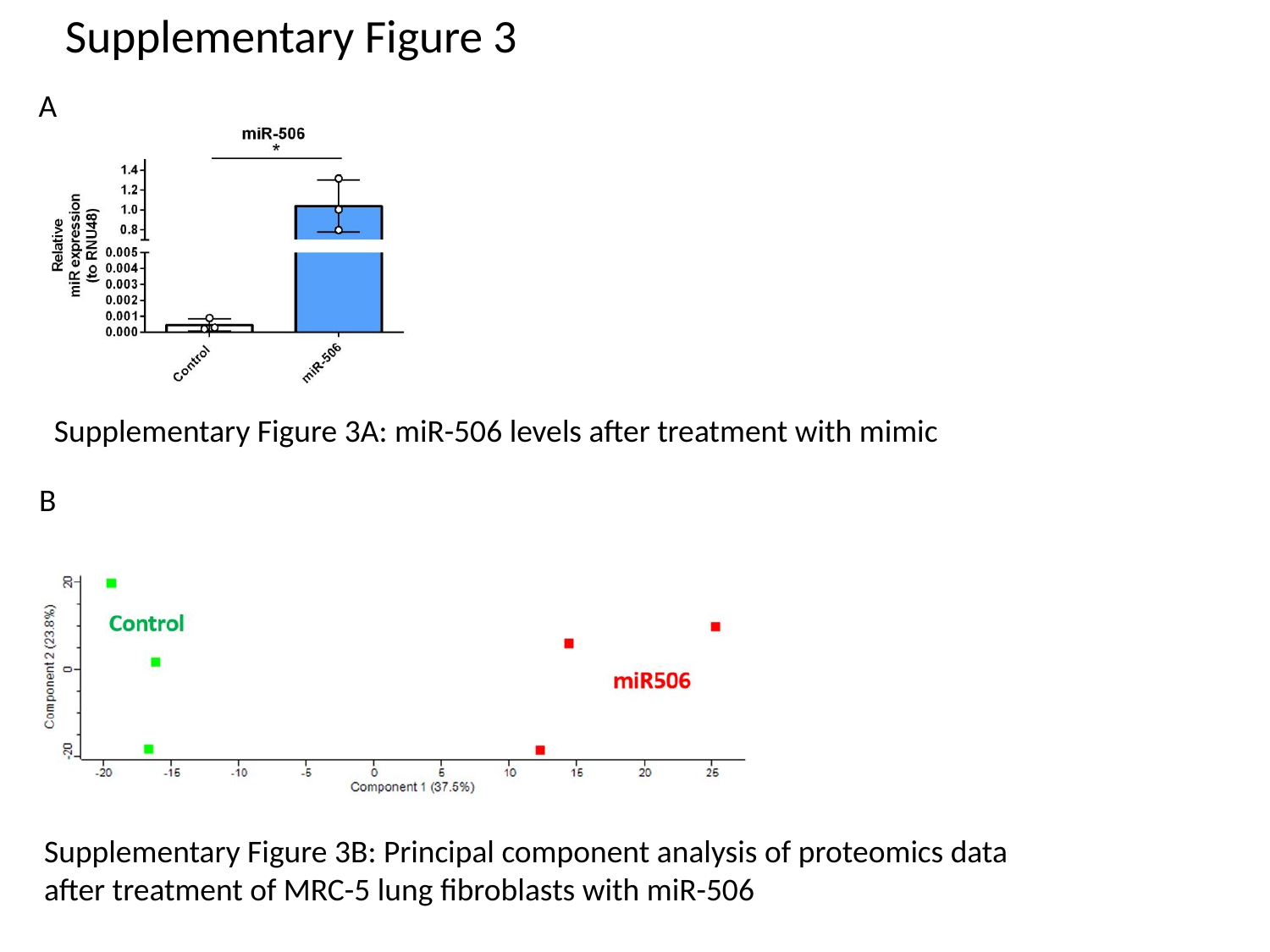

Supplementary Figure 3
A
Supplementary Figure 3A: miR-506 levels after treatment with mimic
B
Supplementary Figure 3B: Principal component analysis of proteomics data
after treatment of MRC-5 lung fibroblasts with miR-506

## Slide 8
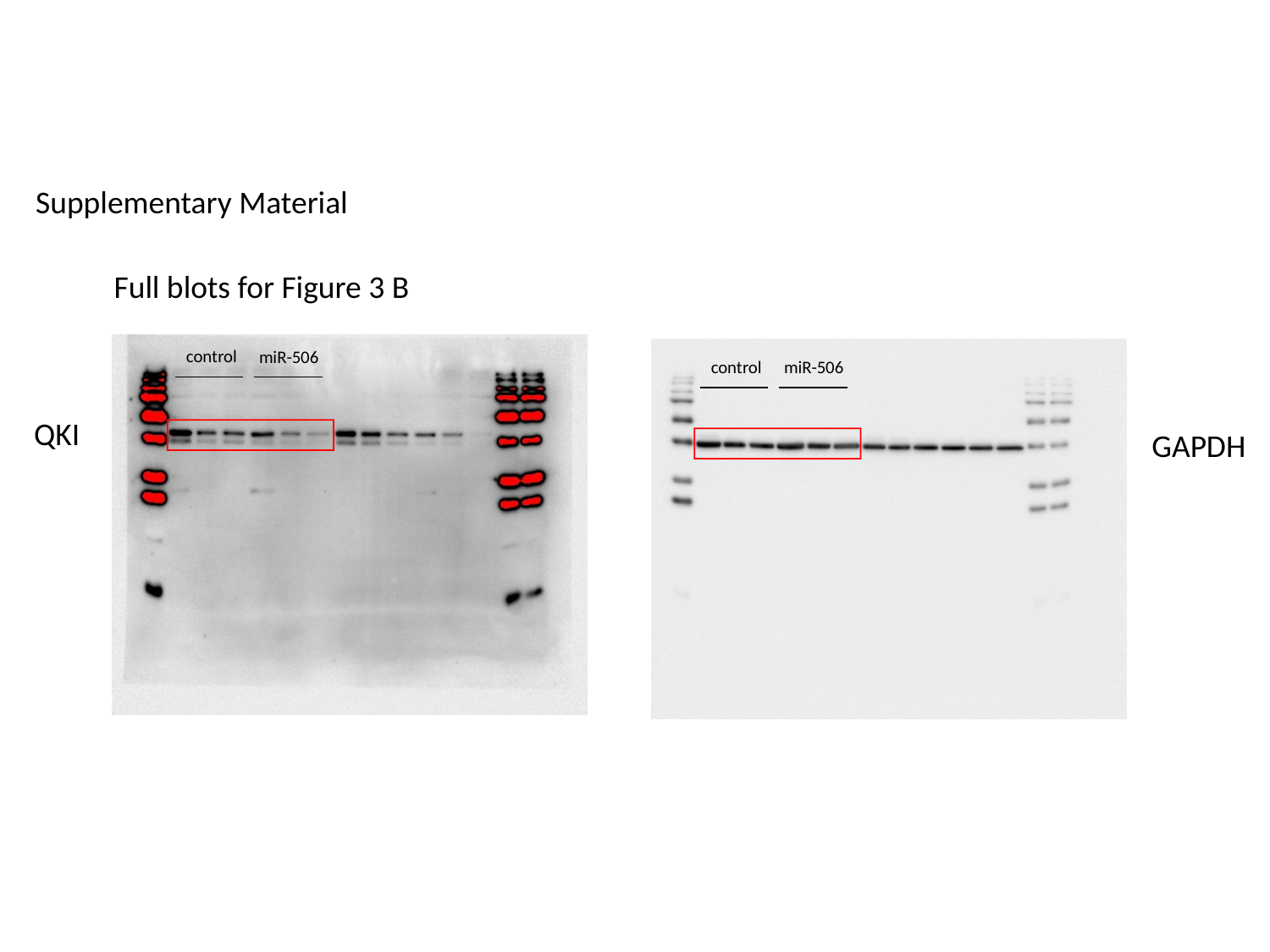

Supplementary Material
Full blots for Figure 3 B
control
miR-506
control
miR-506
QKI
GAPDH

## Slide 9
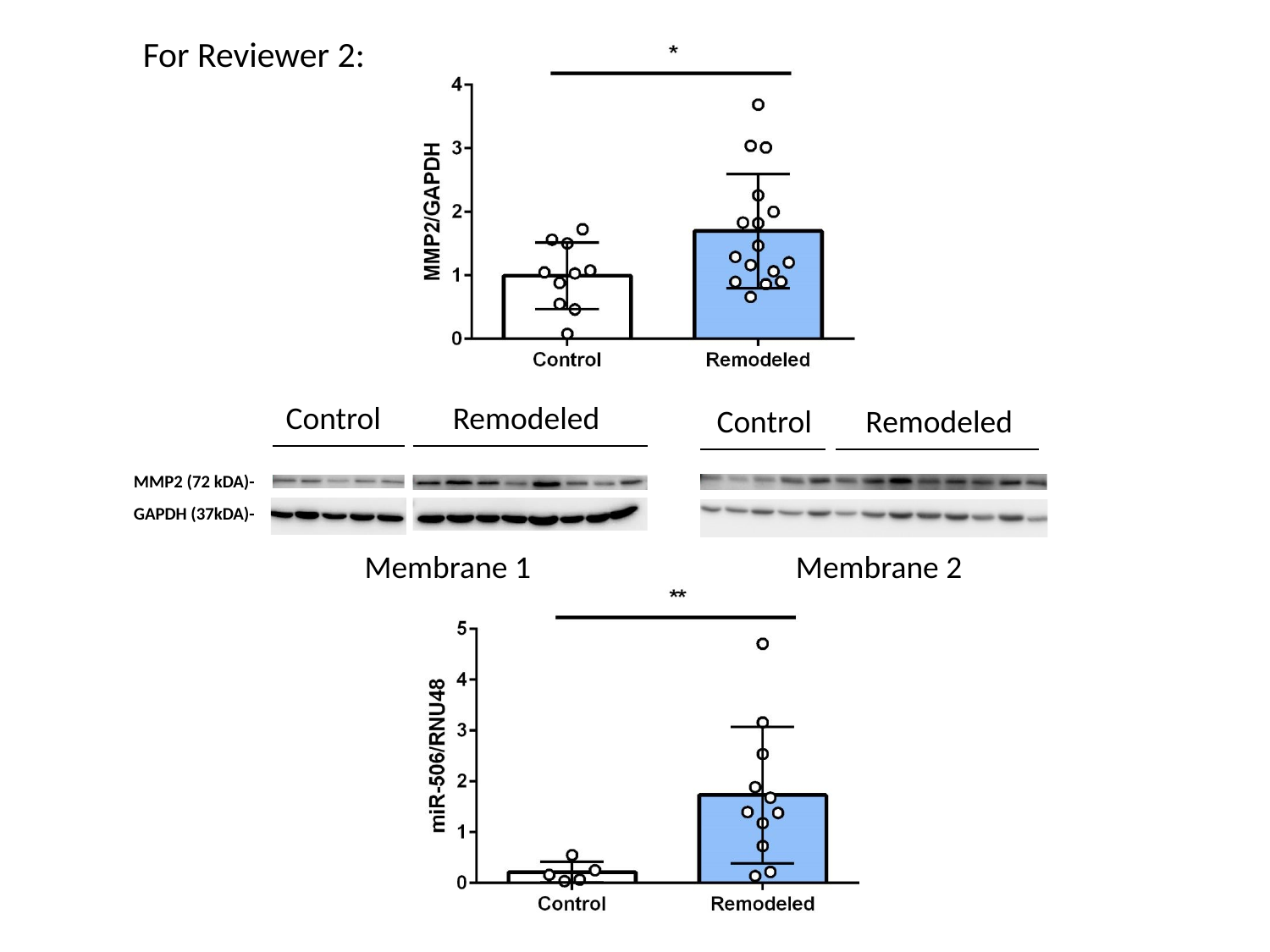

# For Reviewer 2:
Control
Remodeled
Control
Remodeled
MMP2 (72 kDA)-
GAPDH (37kDA)-
Membrane 1
Membrane 2

## Slide 10
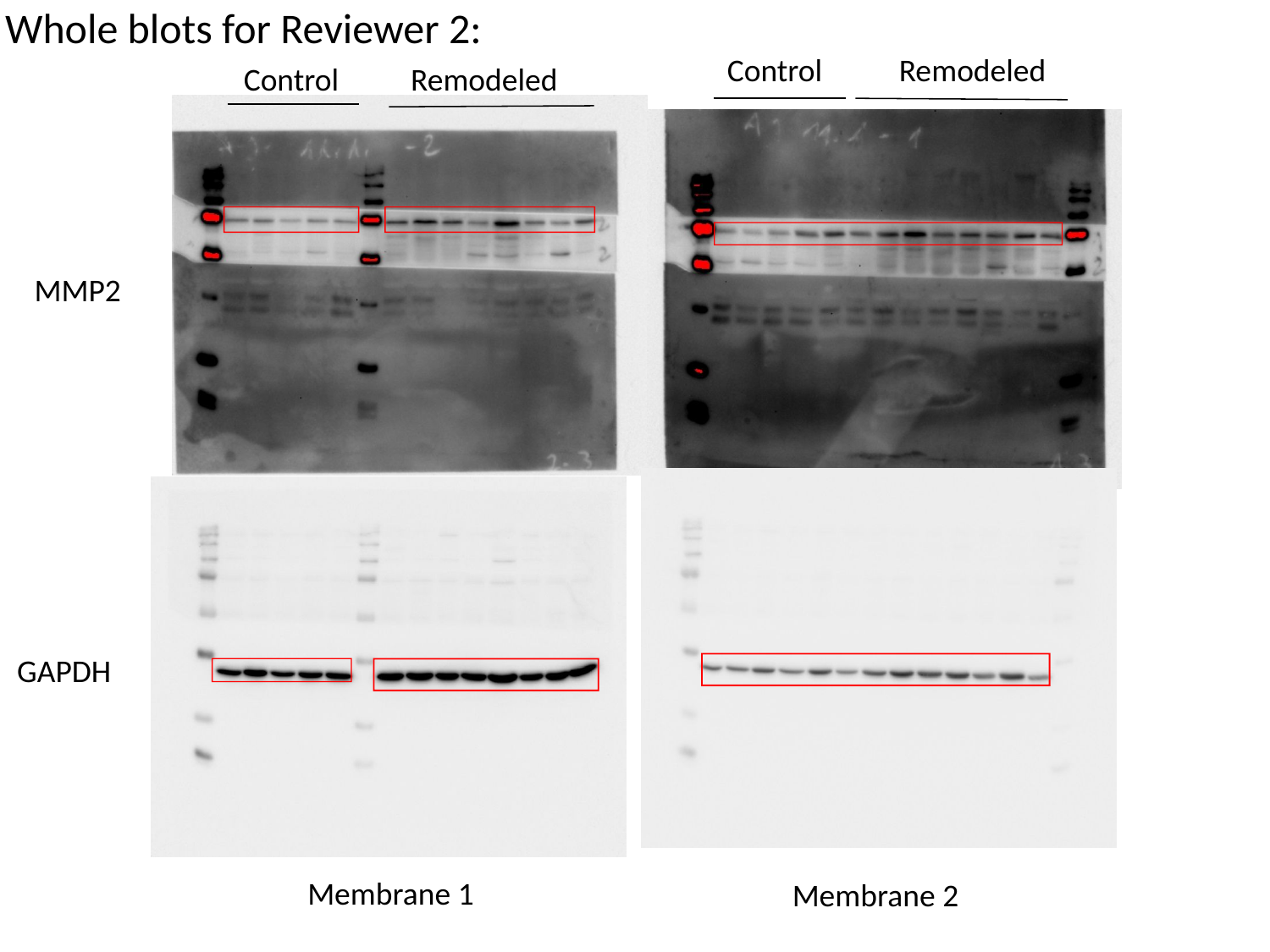

# Whole blots for Reviewer 2:
Remodeled
Control
Control
Remodeled
MMP2
GAPDH
Membrane 1
Membrane 2
